# Pain Recognition With Electrocardiographic Features in Postoperative Patients: Method Validation Study

**DOI:** 10.2196/25079

**Published:** 2021-05-28

**Authors:** Emad Kasaeyan Naeini, Ajan Subramanian, Michael-David Calderon, Kai Zheng, Nikil Dutt, Pasi Liljeberg, Sanna Salantera, Ariana M Nelson, Amir M Rahmani

**Affiliations:** 1 Department of Computer Science University of California, Irvine Irvine, CA United States; 2 Department of Anesthesiology and Perioperative Care UC Irvine Health Orange, CA United States; 3 Department of Informatics University of California, Irvine Irvine, CA United States; 4 Department of Future Technology University of Turku Turku Finland; 5 Department of Nursing University of Turku Turku Finland; 6 Turku University Hospital Turku Finland; 7 School of Medicine University of California, Irvine Irvine, CA United States; 8 School of Nursing University of California, Irvine Irvine, CA United States; 9 Institute for Future Health University of California, Irvine Irvine, CA United States

**Keywords:** pain assessment, recognition, health monitoring, wearable electronics, machine learning

## Abstract

**Background:**

There is a strong demand for an accurate and objective means of assessing acute pain among hospitalized patients to help clinicians provide pain medications at a proper dosage and in a timely manner. Heart rate variability (HRV) comprises changes in the time intervals between consecutive heartbeats, which can be measured through acquisition and interpretation of electrocardiography (ECG) captured from bedside monitors or wearable devices. As increased sympathetic activity affects the HRV, an index of autonomic regulation of heart rate, ultra–short-term HRV analysis can provide a reliable source of information for acute pain monitoring. In this study, widely used HRV time and frequency domain measurements are used in acute pain assessments among postoperative patients. The existing approaches have only focused on stimulated pain in healthy subjects, whereas, to the best of our knowledge, there is no work in the literature building models using real pain data and on postoperative patients.

**Objective:**

The objective of our study was to develop and evaluate an automatic and adaptable pain assessment algorithm based on ECG features for assessing acute pain in postoperative patients likely experiencing mild to moderate pain.

**Methods:**

The study used a prospective observational design. The sample consisted of 25 patient participants aged 18 to 65 years. In part 1 of the study, a transcutaneous electrical nerve stimulation unit was employed to obtain baseline discomfort thresholds for the patients. In part 2, a multichannel biosignal acquisition device was used as patients were engaging in non-noxious activities. At all times, pain intensity was measured using patient self-reports based on the Numerical Rating Scale. A weak supervision framework was inherited for rapid training data creation. The collected labels were then transformed from 11 intensity levels to 5 intensity levels. Prediction models were developed using 5 different machine learning methods. Mean prediction accuracy was calculated using leave-one-out cross-validation. We compared the performance of these models with the results from a previously published research study.

**Results:**

Five different machine learning algorithms were applied to perform a binary classification of baseline (BL) versus 4 distinct pain levels (PL1 through PL4). The highest validation accuracy using 3 time domain HRV features from a BioVid research paper for baseline versus any other pain level was achieved by support vector machine (SVM) with 62.72% (BL vs PL4) to 84.14% (BL vs PL2). Similar results were achieved for the top 8 features based on the Gini index using the SVM method, with an accuracy ranging from 63.86% (BL vs PL4) to 84.79% (BL vs PL2).

**Conclusions:**

We propose a novel pain assessment method for postoperative patients using ECG signal. Weak supervision applied for labeling and feature extraction improves the robustness of the approach. Our results show the viability of using a machine learning algorithm to accurately and objectively assess acute pain among hospitalized patients.

**International Registered Report Identifier (IRRID):**

RR2-10.2196/17783

## Introduction

### Overview

Pain assessment is a critical public health burden and is essential to effective pain management, which is associated with many illnesses [1]. Pain is “an unpleasant sensory and emotional experience expressed in terms of actual or potential tissue damage” [2], according to the most widely accepted definition. Pain is considered to be a subjective experience that is related to each individual in early life through experiences related to injury [3]. Such pain, which is termed acute pain, usually lasts hours, days, or weeks. Acute pain is associated with soft tissue damage, a surgical procedure, or a brief disease process and fosters avoidance of the harmful action in the future and promotes healing by inhibiting activities that might cause further tissue damage [4]. Pain, as a susceptible and ambiguous phenomenon, is difficult to quantify [5], particularly when the patient's own opinion is difficult to reach due to their limited ability to communicate, as in patients under sedation or anesthesia, persons with intellectual disabilities, infants, and patients during critical illness [6]. Uncontrolled pain could cause some serious complications and may evolve into chronic pain. This could cause longer recovery in hospitals and delayed discharge, higher health care costs, and major psychological, financial, and social ramifications for patients [7]. However, overtreatment of pain can also result in adverse effects such as hospital readmission due to poorly controlled pain after discharge or long-term opioid dependence.

The current and “gold standard” pain assessment relies on patient self-reporting with tools such as the Visual Analogue Scale (VAS) and Numerical Rating Scale (NRS). Although these unidimensional models are considered powerful in acute pain assessment, they are rife with deficits given their interactive communication requirement between the patient and nurses, which is a serious problem in noncommunicative patients [8,9]. Such tools rely on nurses’ knowledge, physical assessment skills, and interviewing techniques. It is thus meaningful to develop better tools to assess pain intensity for continuous real-time pain monitoring. Such a tool not only improves the care process of noncommunicative patients but can also benefit other patient populations with timelier treatment, accurate assessment, and reduced monitoring burden on clinicians [10].

State-of-the-art objective pain intensity assessment algorithms consist of analysis of physiological and physical pain indicators, as multiparameter analysis is superior to a singular physiological parameter [11-13]. Objective pain assessment leverages using wearable devices to capture the physiological parameters. Internet-of-Things (IoT) devices, including wearable devices, play a significant role in objective pain monitoring systems [10]. As an example, Vatankhah et al [14] measured and diagnosed pain levels of human using discrete wavelet transform via electroencephalographic signals. These devices are in charge of various real-time health monitoring services as well as continuously processing and analyzing pain intensity levels to classify them automatically and objectively. However, these solutions to date have only been evaluated on healthy volunteers (stimulated pain). This was the motivation to develop a multimodal data set from postoperative adult patients in hospitals to get a better understanding of pain intensity characteristics of real patients. We call the data set UCI iHurt Database (UCI_iHurtDB) [15]. The data set is planned to be released for research purposes and, to the best of our knowledge, is the first comprehensive data set collected from patients suffering from real postoperative pain.

Electrocardiography (ECG) is useful for indicating the perception of pain among all of the physiological signals captured by wearable devices. Heart rate (HR) and heart rate variability (HRV) are the essential parameters that can be derived from ECG, as they are both coupled to autonomic nervous system activity, when internal body functions are involuntarily regulated, and they can provide a suitable proxy for examining pain intensity [16]. The most frequently used vital sign in pain studies is HR as the number of heartbeats, while HRV features with the extent to which the heart rate changes over a time interval or the extent to which it is spread over different frequencies are observed individually and selectively in some pain studies [17-19]. There are several approaches for classifying pain intensity of healthy subjects using machine learning techniques. Lopez-Martinez and Picard [18] explored traditional machine learning algorithms such as logistic regression and support vector machine (SVM) with different kernels, as well as recurrent neural network, to create a model for no pain versus pain at different intensities. Koenig et al [19] revealed that HRV is a promising measure of autonomic reactivity to nociceptive stimulation in healthy adults. Therefore, we examine HR and HRV features with their correlation with pain for future biosignal fusion in pain intensity assessment and pain detection.

To the best of our knowledge, this is the first work to study the relations between ECG physiological signal and pain intensity of postoperative patients to predict different pain intensity levels. This will promote advancements in both observational and physiological pain measurement. The technology used in this paper for objective pain assessment was developed by our group and presented in our previous work by Sarker et al [20]. The prototype device can be variably configured for inclusion in a wide range of applications. It is also validated on healthy adults under thermal and electrical experimental pain stimulus [21].

In this paper, we looked into the ECG signal as one of the physiological signals from the human body and examined HR and HRV features extracted from ECG in several categories to assess their correlation with pain intensity for future physiological data fusion in pain intensity assessment. Due to limited available self-report pain intensity labels, we explored weak supervision, which has shown that performance of the end-model can asymptotically improve with data set size, although noisy sources of supervision are used [22,23]. Thus, the contributions of this work are twofold: (1) We present UCI_iHurtDB, a freshly collected data set from postoperative patients consisting of multimodal biosignals (ECG, electromyography, electrodermal activity, photoplethysmography, accelerometer). (2) We provide a novel weakly supervised method to enhance the sparsely labeled data set. To the best of our knowledge, this is the first study using weak supervision in pain assessment.

### Interpretation of ECG in Pain Studies

Interpretation of ECG in pain studies starts with the ability to detect the QRS complex as one of the morphological parts of the ECG waves, with the focus on the RR intervals (distance between adjacent R-waves). These peaks are essential in HRV analysis, where in literature they are usually referred to as NN intervals. HRV features have been traditionally calculated over a short period of 5 minutes or over a long period in 24 hours. However, in some cases such as acute physiological changes, some HRV features in less than a minute are also taken on as ultra–short-term analysis. Within each time window, HRV features can be extracted from NN intervals in several domains including (1) time domain features and (2) frequency domain features. HRV features in the time domain consist of statistical features including but not limited to SD of NN intervals, SD of average NN intervals, root mean square of successive differences (RMSSD), NN50, pNN50, and Max(HR) − min(HR). HRV features in the frequency domain are extracted by the Welch averaging method using power spectral density.

In literature, HRV analysis has been examined as one of the main ways to measure pain in different types; in Sesay et al [24], for instance, with the majority of 120 patients, it was observed that regarding acute pain after minor surgery, NRS was correlated with low-frequency (LF) band and the ratio of LF to high-frequency (HF) band but not with HF. To monitor the nociception level of patients with multiple physiological parameters, HF in a 1-minute window was calculated in Ben-Israel et al [11]. Jiang et al [17] experimented with the correlation of HRV features in the ultrashort term with acute pain. They suggested that multiple HRV features can indicate the change from no pain to pain. Werner et al [25] compared no pain among pain levels 1-4 using a random forest (RF) classifier. They reported the detection of pain using a set of features from ECG signals.

## Methods

### Setting

The study was approved by the University of California, Irvine (UCI) institutional review board (HS: 2017-3747). Candidates were selected from the Acute Pain Service (APS) patient list at UCI Health in Orange, California. The APS unit at the medical center serves approximately 100 patients weekly, enabling the lead Doctor of Medicine to recruit patients.

### Study Description, Participants, and Recruitment

This study is a prospective observational data collection from postoperative patients likely having mild to moderate pain. All 25 participants recruited for this study met the following criteria: (1) age at least 18 years, (2) received a consult by the APS, (3) able to communicate, (4) able to provide written informed consent, and (5) healthy, intact facial skin. They were excluded if they had (1) any diagnosed condition affecting cognitive functions (dementia, psychosis), (2) any diagnosed condition affecting the central nervous system, facial nerves or muscles, (3) deformities on hand that prevent sensor placement, or (4) significant facial hair growth in the area where the sensors were going to be attached.

Potential participants were selected if they were determined to be eligible to participate in this study based on the aforementioned inclusion and exclusion criteria. Patients got both oral and written information about the details of the study. Candidates were provided at least 24 hours to consider participation in the study before finalizing the consent form, and they were recruited to participate in this study after obtaining the written consent form.

### Study Design

After obtaining the written consent form, approximately 30 minutes of continuous ECG data was collected from the patients in their private room using multiple wearable sensors in two parts. In the first part, we used a transcutaneous electrical nerve stimulation (TENS) unit to obtain the baseline of the person. Patients were asked to increase the intensity of the TENS device up to the level that was tolerable for them, hold it for at least 10 seconds, and then decrease it to level 0, including additional rest between TENS challenges. In the second part, patients were engaged with soft activities such as walking, coughing, sitting, and lifting legs that may cause pain sensation. To achieve a better statistical analysis, data collection in both parts, with and without TENS unit, was repeated two to three times. Subject's self-report of pain was recorded using NRS. The NRS for pain is a segmented numeric version of the VAS to measure pain intensity in one dimension in which a respondent points to the number on the NRS, an integer from 0 to 10, that best represents their pain intensity from “no pain” to “worst pain” [26]. We expect to find solutions from multiple parameters that are robust in response to different acute pain cases or study designs.

### Data Collection

To develop an algorithm for pain assessment in hospitalized patients, we tracked physiological signals such as HR, HRV, and respiratory rate from their ECG signals. The technology used in this study to capture the aforementioned signals includes the following components:

1. Biopotential acquisition system for ECG recording—ECG is a biopotential signal captured from the skin surface with a device developed by Sarker et al [20]. The system includes commercially available electrodes (eg, in 24 mm diameter), electrode-to-device lead wires, an ADS1299-based portable device, and computer software receiving streaming data from the portable device. The raw data of each channel at the rate of 500 samples per second is sent to the computer software through Bluetooth. The software visualizes the waveforms and saves the raw data into files. The common reference electrode is placed on the neutral bony area behind the ear. This device uses two channels to collect 2-lead ECG. One channel is to measure the potential between ECG - right arm and reference, and the other channel is to measure the potential between ECG - left arm and reference.
2. TENS unit device (Food and Drug Administration–cleared Class II over-the-counter HealthmateForever YK15AB electrotherapy device)—TENS units work by delivering small electrical impulses through electrodes that have adhesive pads to attach them to a person's skin.

### Data Preprocessing

#### Data Synchronization

The ECG signals from each patient were sampled at a rate of 500 Hz. Data from two channels (left arm, right arm) were obtained. The patient’s pain levels were simultaneously reported and saved as labels. For the purpose of synchronicity, the corresponding Unix timestamps were also obtained while extracting both ECG and label data. The ECG signals were trimmed from start to end to match the corresponding label timestamps. Since the sampling frequency was 500 Hz, each timestamp had 500 ECG samples associated with it.

#### Peak Detection

Once these clean signals were obtained, the second step in the pipeline was to extract peaks. To find the peaks, the signals were first sampled down to 250 Hz. A bidirectional long short-term memory network was used to obtain the probabilities and locations of peaks [27]. A window size of 1000 samples and stride of 100 samples was used to generate these predictions. Mean values were obtained from predictions that came from overlapping windows. The predictions that were below a particular threshold (0.05) were discarded and filtered out. Only those peaks that were in local maximum were selected. Once the peaks were obtained, the signal was resampled back to 500 Hz, and the peak probabilities and locations were obtained. This method, however, might still be susceptible to false positives that are likely generated due to the presence of noise or irregular heartbeats. Therefore, another preprocessing step that removes peaks that occur too close to each other was employed. A rolling window was used to remove peaks that occurred in a time period of 450 milliseconds or less between neighboring peaks. The final selected peaks were then appended with their corresponding Unix timestamps. This process was repeated for every patient.

#### Noise Removal

The third and final preprocessing step is to remove noise from the NN interval data. NN intervals are the time intervals between two successive peaks. They are obtained by subtracting two successive peak indices. All data points that are within 2 standard deviations of the mean were selected. The rest of the data points were considered outliers and were removed. Even after removing these outliers, however, there might still be anomalous (not a number [NaN]) values in noisy sections of the data. If the proportion of NaN values exceeded 50 percent, the noisy sections were discarded. Otherwise, only NaN values were discarded, and the remaining values were interpolated. The filtered NN intervals were then saved and used for feature extraction.

### Feature Extraction

In the experiments conducted by Werner et al [25], they used 5.5 seconds of ECG signals and extracted 3 time domain HRV features from the ECG signal of each subject: (1) the arithmetic mean of time in between consecutive heartbeats or mean of NN intervals (AVNN), (2) RMSSD, and (3) the slope of the linear regression of NN intervals or the measure of acceleration of the heart rate. We use an open-source Python toolbox named pyHRV [28] to compute HRV features in our feature extraction process.

In addition to the 3 time domain features used by Werner et al [25], we also computed a few other time domain and frequency domain features. For classification, we conducted two separate experiments: (1) with the three above features only and (2) with additional time and frequency domain features using feature selection. The additional extracted features are mentioned below.

#### Time Domain Features

There were 19 time domain features extracted from the NN interval series. They include the slope of the ECG signals and 18 statistical measures. These features were computed using 5.5-second sliding windows. The definitions of these time domain features are mentioned in Table 1.

**Table 1:** Time domain heart rate variability features and their definitions.

Feature	Description
HR^a^ (ms)	Beats per minute
AVNN (ms)	Mean of NN intervals
SDNN (ms)	Standard deviation of NN intervals
RMSSD (ms)	Root mean square of successive NN interval differences
NNXX (ms)	Number of NN interval differences greater than the specified threshold
pNNXX (%)	Percentage of successive NN intervals that differ by more than XX ms

^a^HR: heart rate.

The breakdown of the 19 aforementioned features is explained as follows: slope of NN intervals—a polynomial fit of degree 1; 5 NN interval features—total count, mean, minimum, maximum, SD; 9 NN interval difference features—mean difference, minimum difference, maximum difference, SD of successive interval differences, root mean square of successive interval differences, number of interval differences greater than (a) 20 milliseconds and (b) 50 milliseconds, and percentage of successive interval differences that differ by more than (a) 20 milliseconds and (b) 50 milliseconds; and 4 heart rate features—mean, minimum, maximum, and SD.

#### Frequency Domain Features

There were 13 frequency domain features extracted by estimating of power spectral density using the Welch method. These features were computed using 250-second rolling windows with a minimum threshold of 50 values per window. The definitions of these frequency domain features are mentioned in Table 2.

**Table 2:** Frequency domain heart rate variability features and their definitions.

Feature	Description
VLF power (s^2^)	Absolute power in very low-frequency band (≤0.04)
LF power (s^2^)	Absolute power in low-frequency band (0.04-0.15)
HF power (s^2^)	Absolute power in high-frequency band (0.15-0.4)
LF peak (Hz)	Peak frequency in low-frequency band (0.04-0.15)
HF peak (Hz)	Peak frequency in high-frequency band (0.15-0.4)
Total power (s^2^)	Total power over all frequency bands
LF/HF (%)	Ratio of LF-to-HF power

The breakdown of the 13 frequency domain features is explained as follows: total power—total spectral power over all frequency bands; 4 HF band fast Fourier transform (FFT) features—peak, absolute, relative, normalized; 4 LF band FFT features—peak, absolute, relative, normalized; 3 very low-frequency (VLF) band FFT features—peak, absolute, relative; and FFT ratio of HF and LF bands.

### Feature Selection

To ensure generalization and avoid overfitting, it is important to perform feature selection. This, in turn, reduces computational complexity and the time for training and validating models. Feature selection models can be placed under three broad categories: filter-based methods, wrapper-based methods, and embedded methods. Filter-based methods statistically determine the relationship between input variables (features) and the target variable (label). They provide a metric for evaluating and filtering out features that will be used by the model. They are also computationally cheaper than the other two methods and have a reduced risk of overfitting [29]. Among the filter-based methods, Gini impurity/information gain is a widely used method to select the most informative features for a classification problem. Usually, a decision tree–based model like an RF classifier is used to output a feature importance vector. Every node of a decision tree represents a condition on how to split values present in a single feature. In this process, similar data on the condition variable end up on the same side of the split. The splitting condition is based on impurity of the features chosen in every node. During the training process, how much each feature contributed to the decrease in impurity is calculated, and features are then ranked based on this measure.

### Labels

#### Label Distribution

Table 3 shows the distribution of NRS pain labels reported by patients during the clinical trials.

**Table 3:** Numerical Rating Scale distribution for 11 pain classes.

Reported Numerical Rating Scale labels	n
0	37
1	52
2	37
3	61
4	83
5	44
6	32
7	16
8	46
9	26
10	4

Since the NRS labels recorded during clinical trials were collected from real postoperative patients, there are some inherent challenges due to the distribution of data. For example, there are 83 occurrences of NRS pain label 4, but there are only 4 occurrences of NRS pain label 10 among all patients. Due to the subjective nature and the different sources of pain among our recruited patients, the imbalanced distribution of pain levels among all patients is inevitable.

To compare our pain assessment algorithm’s performance with Werner et al [25], we downsampled our pain labels from 11 NRS classes (0-10) to 5 classes (0-4). Data points from NRS pain label 0 were considered as a baseline, and the remaining NRS pain labels were distributed among 4 classes. Thresholds for each downsampled class were carefully chosen in order to minimize an imbalanced class distribution. Table 4 shows the resulting distribution after downsampling the NRS pain labels. The relatively large number of occurrences of NRS pain label 4 increased the number of downsampled PL2 labels over other downsampled pain labels.

**Table 4:** Downsampled pain distribution with 5 classes.

Downsampled pain labels	n
BL^a^	37
PL1^b^	89
PL2	144
PL3	92
PL4	76

^a^BL: baseline.

^b^PL: pain level.

#### Labeling ECG Features

Since the patients’ NRS values were only reported after performing some activities, labels were stored sparsely. While combining ECG features with their corresponding labels, their timestamps were matched based on the nearest 5.5 seconds (labeling threshold). More precisely, any ECG feature window that was within 5.5 seconds of a reported NRS value was given that value as its label. However, as a consequence of this, all the feature windows that were not within the labeling threshold were not given a corresponding label.

**Figure 1:**
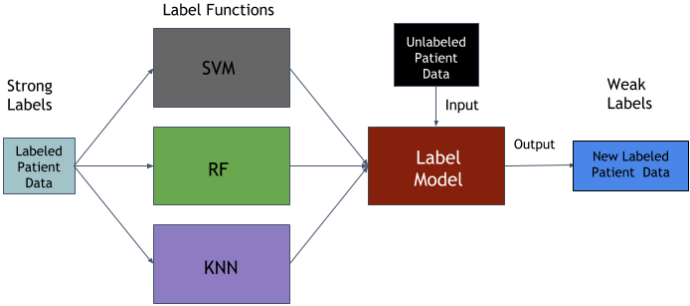
Snorkel labeling architecture. KNN: k-nearest neighbor; RF: random forest; SVM: support vector machine.

To label the remaining unlabeled data points, we employed Snorkel [23], a weak supervision framework for rapid training data creation. Snorkel is an end-to-end system that combines weak supervision sources to label training data with limited ground truth data. Rather than hand-labeling training data, Snorkel allows its users to write labeling functions that make use of heuristics, patterns, third-party machine learning models, external knowledge bases, and more. Weak supervision is typically employed to label large amounts of unlabeled data when there are noisy, limited, or imprecise sources. This approach eliminates the burden of continuously obtaining NRS values from patients. The use of Snorkel in our labeling process allowed us to make use of more data for the purpose of training and testing our pain algorithm. This consequently led to better performance during validation. Figure 1 depicts the architecture that was used to label these unlabeled instances.

All the data points that were within the labeling threshold were considered “strong” labels, or labels used for training labeling functions. These strong labels were collected directly from the patients. The remaining unlabeled data points were considered “weak” data and were kept aside for the weak supervision algorithm to label. For the labeling process, only each patient's strongly labeled data was used to label their own unlabeled instances. This was done to avoid the possibility of data leakage during the validation process.

The labeling functions consist of a group of three off-the-shelf machine learning models: (1) an SVM with a radial basis function kernel, (2) an RF classifier, and (3) a k-nearest neighbor (KNN) classifier with uniform weights. Once each of these models is trained on strong labels, they are used to make predictions on the weak or unlabeled data. Their predictions are then collected and converted into a single confidence-weighted label per data point using Snorkel’s *LabelModel* function. The most confident label predictions from each datapoint were considered as labels for the weak data. It is important to note that weak supervision does not compromise on the reliability of our algorithm because we use the weakly labeled data only for training our models. This way, the performance of our algorithm can be measured using only real data collected from patients.

### Classification

To compare the performance of our pain algorithm with Werner et al [25], we performed binary classification on our test data using the 3 time domain features mentioned in their work. We split the binary classification problem into 4 different categories: baseline (BL) versus pain level 1 (PL1), BL versus PL2, BL versus PL3, and BL versus PL4. Since 1 of the patients had data from only one downsampled label class, they were discarded from the classification process. Consequently, we were left with data from 19 patients.

We evaluated the performance of our pain algorithm using leave-one-out cross-validation (LOOCV) with the focus on optimizing the area under the curve (AUC) score. During each iteration of LOOCV, the data of 18 of the 19 patients, including those data points that were labeled by Snorkel, were used for training. For testing, only the strongly labeled data points from the one patient left out were used. This process is repeated for all 19 patients to estimate the algorithm’s performance on unseen data. Due to the presence of an imbalanced distribution of pain levels within patients, data points from some pain levels were nonexistent from their data. As a result, it was not possible to compute either precision or recall for most patients.

The following five classification methods were deployed in our experiments to identify the best performing model for our pain assessment algorithm: AdaBoost classifier, XGBoost classifier, RF classifier, SVM classifier, and KNN classifier.

We also conducted separate experiments with feature selection using the 32 features mentioned in the Feature Extraction section. To get the best set of features for classification, we run LOOCV using an RF classifier. We compute the Gini importance of each of the features at every fold and select those features that were at least one standard deviation above the mean importance score. As a result, it was possible to have different sets of features in every fold. After computing the best set of features at every fold, we consider those features that were used in most of the folds for classification. The following 8 features were used in the final feature set: (1) total spectral power, (2) absolute LF power, (3) absolute HF power, (4) mean HR, (5) relative HF power, (6) normalized HF power, (7) relative VLF power, and (8) normalized LF power.

## Results

### Pain Demographic Characteristics

A total of 25 patients with acute pain were engaged by APS and recruited for this study at UCI Medical Center. However, the ECG data from 2 patients were missing due to connectivity issues. Moreover, we found that 3 of the patients had arrhythmia, so we removed those 3 as well. The average age of patients was 55.6 years (SD 16.24, range 23-89); 52% (13/25) of patients were male and 48% (12/25) of patients were female (Table 5). All of the patients (n=20) were taking prescription medication at the time of the study. The nature of the procedures for each participant included the following domains: 50% general surgery (diagnostic laparoscopy, exploratory laparotomy, and vascular), 25% orthopedics, 15% trauma (thoracic pain and rib plating), and 10% urology (cystectomy and bladder augmentation).

**Table 5:** Patient demographic characteristics (N=25).

Variable		Value	Range
Patients excluded due to arrhythmia, n (%)		3 (12)	N/A^a^
Patients excluded due to missing ECG^b^ data, n (%)		2 (8)	N/A
Gender, male, n (%)		13 (52)	N/A
Weight (kg), mean (SD)		76.56 (17.31)	52.2-112.2
Height (cm), mean (SD)		170.9 (10.44)	152.4-193
BMI^c^ (kg/m^2^), mean (SD)		26.33 (6.14)	15.1-38.73
**Procedure domain (n=20), n (%)**			
	General surgery	10 (50)	N/A
	Orthopedics	5 (25)	N/A
	Trauma	3 (15)	N/A
	Urology	2 (10)	N/A

^a^N/A: not applicable.

^b^ECG: electrocardiography.

^c^BMI: body mass index.

### Pain Engagement Results

**Figure 2:**
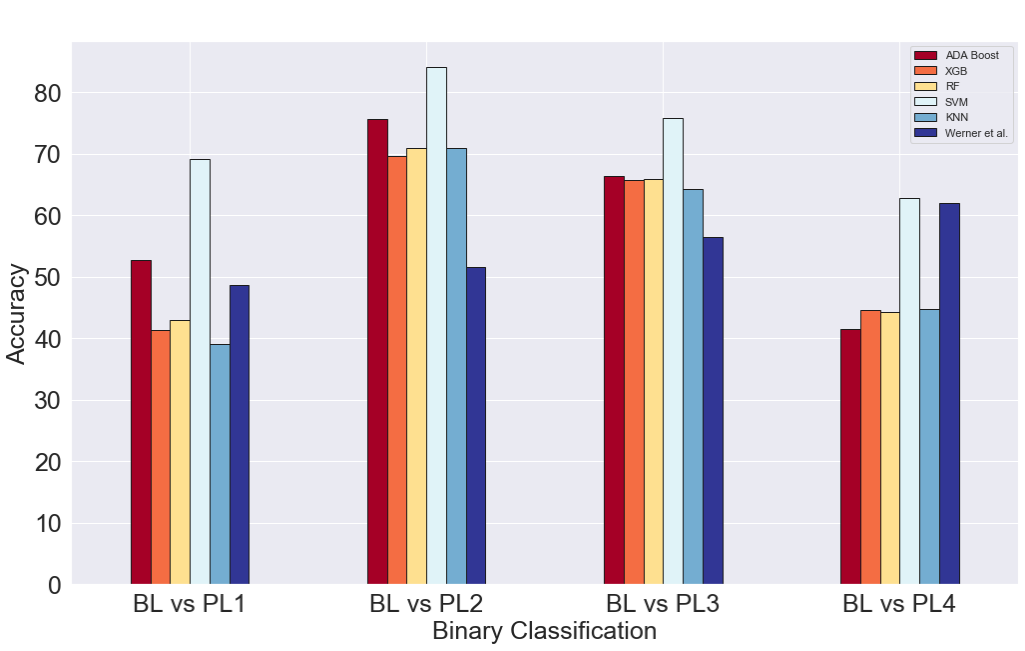
Validation accuracy of all classifiers on BioVid features. BL: baseline; PL: pain level; KNN: k-nearest neighbor; RF: random forest; SVM: support vector machine; XGB: XGBoost.

**Figure 3:**
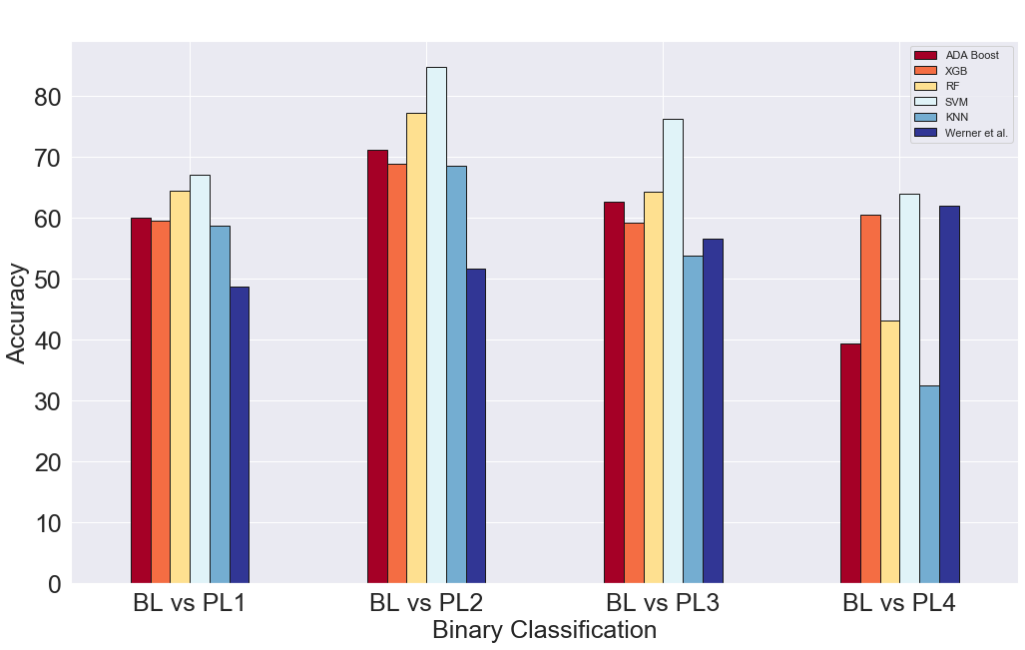
Validation accuracy of all classifiers on top 8 features. BL: baseline; PL: pain level; KNN: k-nearest neighbor; RF: random forest; SVM: support vector machine; XGB: XGBoost.

To make a fair comparison between our pain assessment algorithm and the work of Werner et al [25], we replicated their settings into our data set. The comparisons of the accuracy achieved by our algorithm on all five classifiers while using only 3 time domain features are shown in Figure 2. Similarly, Figure 3 shows the same comparison while performing feature selection. These figures show the mean accuracy across all subjects while performing 4 different binary classifications based on pain levels. The final scores are presented in Table 6 and Table 7 below.

**Table 6:** Validation accuracy of BioVid features.

Binary classification	AdaBoost	XGBoost	RF^a^	SVM^b^	KNN^c^	Werner et al
BL^d^ vs PL1^e^	52.63	41.35	42.97	69.16	39.06	48.7
BL vs PL2	75.68	69.57	70.84	84.14	70.92	51.6
BL vs PL3	66.33	65.73	65.94	75.73	64.20	56.5
BL vs PL4	41.53	44.55	44.24	62.72	44.68	62.0

^a^RF: random forest.

^b^SVM: support vector machine.

^c^KNN: k-nearest neighbor.

^d^BL: baseline.

^e^PL: pain level.

**Table 7:** Validation accuracy of top 8 features.

Binary classification	AdaBoost	XGBoost	RF^a^	SVM^b^	KNN^c^	Werner et al
BL^d^ vs PL1^e^	59.94	59.46	64.37	67.03	58.61	48.7
BL vs PL2	71.06	68.85	77.19	84.79	68.54	51.6
BL vs PL3	62.63	59.22	64.29	76.18	53.76	56.5
BL vs PL4	39.29	60.44	43.17	63.86	32.51	62.0

^a^RF: random forest.

^b^SVM: support vector machine.

^c^KNN: k-nearest neighbor.

^d^BL: baseline.

^e^PL: pain level.

We were able to achieve the highest accuracy on the SVM classifier for both settings, with and without feature selection. Moreover, there is no noteworthy difference in the performance of the SVM classifier in both settings. However, while comparing the other classifiers, it is evident that there is a great improvement in performance while using feature selection in the BL versus PL1 category. The performances of the AdaBoost, XGBoost, RF, and KNN classifiers have a marked increase of about 12% on average when compared to their counterparts without feature selection. However, there is a slight decrease in the AdaBoost and RF classifiers and significant decrease in the KNN performance in the BL versus PL4 category. On the other hand, there is an improvement of about 16% in the XGBoost classifier while performing feature selection in the BL versus PL4 category. We speculate that the lower accuracy scores could be due to the relatively smaller number of training examples available from the downsampled PL4. On the flip side, due to the relative abundance of training examples from PL2, there is a spike in performance for all classifiers across both feature settings in the BL versus PL2 category.

While comparing our algorithm's performance to Werner et al [25], we can see that our SVM classifier fares significantly better than their model. The SVM classifier outperforms their model by an average of 20% across both feature settings for the first three pain categories (BL vs PL1, BL vs PL2, and BL vs PL3). Conversely, there is only a slight increase in performance across both feature settings in the BL versus PL4 category.

## Discussion

### Strengths

To the best of our knowledge, this is the first study that uses ECG signals from real postoperative adult patients for the purpose of developing an automatic pain assessment tool. Moreover, the use of weak supervision in our data labeling process is a novel approach that has not been implemented in pain assessment studies before. It eliminates the need for constantly asking patients for their pain levels and therefore reduces the burden placed on them during the trials. The accuracy scores for this data set with the focus of optimizing it for AUC using the SVM classifier, especially in the first three pain categories, are considerably higher than the scores achieved by Werner et al for both feature settings (with and without feature selection). We also achieve comparable results for the last pain category (BL vs PL4). Furthermore, the use of feature selection in our procedure helps determine the most informative features and reduces the complexity of our pain models. We were able to identify the 8 most informative features and improve the performance of our models in the process.

### Limitations

The main limitation in our algorithm is the presence of noise, in the form of motion artifacts, in our physiological data. Since we collect data from real postoperative patients in a clinical setting, they were allowed to move more freely when compared to experiments performed in a laboratory setting on healthy subjects. The presence of noise diminished the quality of our data. Thus, this negatively impacted the performance of our algorithm. 

Another limitation in our experiments is the presence of imbalanced labels in each patient’s data. Since we did not collect data in a laboratory setting, most patients did not report all the different pain levels during the trials. Most noticeably, this led to a relatively smaller number of labeled examples from the highest pain level (PL4). This consequently decreased the performance accuracy for that pain category (BL vs PL4). In a controlled laboratory setting, one can design the study to force the pain intensity levels to be balanced, which is not feasible in real settings.

Furthermore, we could not find a significant difference between different pain levels in our study. We believe this is due to the fact that variations in ECG signals in response to different pain levels are much harder to distinguish in comparison to different pain levels versus baseline. Moreover, it is worth mentioning that the state of the art in pain assessment focuses on comparing baseline with other pain levels (eg, Werner et al [25]). We believe the reason is to find out if the patient has pain (baseline vs other pain levels).

### Conclusions

The experiments proposed in this study show the viability of our pain assessment algorithm on data from postoperative patients. The use of weak supervision for labeling and feature extraction improves the robustness of our approach. We plan to incorporate multimodal pain assessment methods to further improve our performance and robustness.

## Abbreviations

APS: Acute Pain Service
AUC: area under the curve
BL: baseline
ECG: electrocardiography
FFT: fast Fourier transform
HF: high-frequency
HRV: heart rate variability
IoT: Internet of Things
KNN: k-nearest neighbor
LF: low-frequency
LOOCV: leave-one-out cross-validation
NaN: not a number
NRS: Numerical Rating Scale
PL: pain level
RF: random forest
RMSSD: root mean square of successive differences
SVM: support vector machine
TENS: transcutaneous electrical nerve stimulation
UCI: University of California, Irvine
VAS: Visual Analogue Scale
VLF: very low-frequency
